# The gut hormone glucagon-like peptide-1 produced in brain: is this physiologically relevant?

**DOI:** 10.1016/j.coph.2013.09.006

**Published:** 2013-12

**Authors:** Stefan Trapp, James E Richards

**Affiliations:** Department of Neuroscience, Physiology and Pharmacology, University College London, London WC1E 6BT, UK

## Abstract

Glucagon-like peptide-1 (GLP-1) is both a peripherally expressed incretin and a centrally active neuropeptide. Brain derived GLP-1, produced in preproglucagon (PPG) neurons located in the nucleus of the solitary tract (NTS) and projecting to numerous brain regions, is ideally placed to activate central GLP-1 receptors in a range of autonomic control areas. *In vivo* analysis of central GLP-1 using GLP-1 receptor antagonists has demonstrated the control of a range of feeding responses mediated by GLP-1 receptor activation. Recent advances enabling identification and targeting of the neurons in the NTS has specifically implicated PPG neurons at the core of GLP-1 dependent central and peripheral control for short-term and long-term energy balance.

## Highlights

• PPG neurons express GLP-1 and project to autonomic control sites throughout the brain.
• The distribution of PPG axon terminals mirrors the distribution of GLP-1 receptor cells throughout the CNS.
• Brain-derived GLP-1 plays a role in suppression of hedonic and metabolic food intake.

**Current Opinion in Pharmacology** 2013, **13**:964–969

This review comes from a themed issue on **Endocrine and metabolic diseases**

Edited by **Frank Reimann** and **Fiona M Gribble**

For a complete overview see the Issue and the Editorial

Available online 24th September 2013

1471-4892/$ – see front matter, © 2013 Elsevier Ltd. All rights reserved.

**http://dx.doi.org/10.1016/j.coph.2013.09.006**

## Introduction

‘Gut hormones’ have increasingly been implicated in brain function [1,2]. One such example is glucagon-like peptide-1 (GLP-1), which in addition to being gut-derived, is also synthesised by preproglucagon (PPG) neurones in the brain. These are located primarily in a discrete region of the lower brainstem [3]. This review focuses on the recent advances in our understanding of the physiological significance of this cell population.

The vast majority of studies examining central GLP-1 effects have been performed on rodents and consequently we will focus on these. Although a small number of studies on the GLP-1 system have highlighted differences between mouse and rat [4,5] for most aspects they appear to be equivalent, and in this review we have treated studies on either as comparable. Furthermore, given that the amino acid sequence of GLP-1 is conserved throughout mammalian species [6] and that the distribution of PPG neurons in the non-human primate *Macaca mulatta* [7] is strikingly similar to that in rodents we make the assumption that most of these findings have clear relevance for GLP-1 action in man.

At present it is still controversial as to how the central GLP-1 system is linked to peripheral post-prandial GLP-1 release and whether gut-derived GLP-1 can enter the brain to a sufficient extent to activate central GLP-1 receptors (reviewed in [3,8,9]). Here, we will not address these issues, but consider the GLP-1 producing neurons as an independent cell population and examine the evidence as to the feasibility of the hypothesis that this cell population is the principal physiological source of endogenous GLP-1 interacting with GLP-1 receptors within the CNS. For this to be the case, there has to be anatomical evidence that the distribution of PPG cell axons and GLP-1 release sites matches the distribution of GLP-1 receptors in brain, functional evidence of endogenous release of GLP-1 within the CNS, and proof that inhibition or destruction of these PPG neurons prevents the central effects attributed to GLP-1. We examine these points in sequence.

### Anatomical correlation between GLP-1 receptor expression and the distribution and projections of PPG neurons in the brain

It has been known for more than 20 years that GLP-1 is synthesised in mammalian brain [10–13]. Most published reports analysing the distribution of PPG neurons are from rat using either immunocytochemistry for GLP-1 or GLP-2 [3,10,14–16] or *in situ* hybridisation [17] to localise these neurons and their axon terminals. These studies demonstrated that PPG neurons are non-adrenergic neurons with their cell bodies located exclusively in the caudal nucleus of the solitary tract (NTS), the caudal medullary reticular formation and the olfactory bulb [14,17]. They also demonstrated a widespread projection pattern for these neurons with the highest density of terminals observed in the paraventricular nucleus (PVN) and the dorsomedial hypothalamus (DMH) [14,15,18]. Merchenthaler and colleagues [17] also concluded that all GLP-1 terminals outside the olfactory bulb must originate from the brainstem nuclei, because the olfactory bulb PPG neurons were located periglomerular, and were thus local interneurons. Two recent studies targeting the rostral forebrain confirmed by injection of *Fluoro-Gold* or *RetroBeads* into the nucleus accumbens (NAc) that GLP-1 immunoreactive neurons in the NTS project to this area [19^••^,20^••^].

Recently, Llewellyn-Smith and co-workers [21^•^,22^•^] have revisited the expression pattern of PPG neurons with the use of a transgenic mouse (PPG-YFP mouse) that expresses YFP under the control of the glucagon promoter [23^•^]. These mice show strong YFP fluorescence throughout the entire cytoplasm of the GLP-1 neurons and thus allowed the researchers to map the PPG neurons in mouse with unprecedented precision, showing not only cell bodies and terminals, but also the entire dendritic tree and axons [21^•^,22^•^]. These studies demonstrated mouse PPG cell bodies in the caudal NTS, the intermediate reticular formation mediodorsal of the nucleus ambiguus and along the midline ventral of the hypoglossal nucleus. Additionally, these mice have PPG neurons in the lumbar sacral spinal cord [24] and the granule cell layer of the olfactory bulb [25]. As has been suggested for the olfactory bulb PPG neurons in rat, these appear to be granule cells and thus local interneurons. Consequently, it is expected also in mouse, that all PPG cell projections, which are primarily to autonomic control areas, originate from brainstem PPG neurons. Tracing studies in rat thus far suggest no functional segregation between NTS and reticular PPG neurons [15,18].

In the absence of reliable antibodies for the GLP-1 receptor, the targets for GLP-1 in the brain have been mapped in rat either by identifying GLP-1 binding sites [26] or by *in situ* hybridisation for GLP-1 receptor mRNA [17]. GLP-1 receptors are found throughout the entire rostrocaudal extent of the CNS, from the olfactory bulb down to lamina 5–10 in the sacral spinal cord [17]. However, notable exceptions are cerebral cortex and cerebellum which are devoid of GLP-1 receptors. Interestingly, GLP-1 neurons do not project to these brain structures in rat or mouse [10,14,22^•^]. This good correlation between the expression of GLP-1 receptors and the presence of fibres from GLP-1-expressing neurons is also seen at a regional level; for example, within hypothalamus the arcuate nucleus receives many GLP-1-positive fibres and expresses high levels of GLP-1 receptor, whereas the neighbouring ventromedial nucleus has low levels of both [17,22^•^]. Similarly, Manton *et al.* [24] have recently reported that GLP-1 axons are found throughout the entire rostrocaudal extent of the ventral spinal cord in the PPG-YFP mouse with the highest density of terminals in lamina X and the intermediolateral nucleus (IML). These data correlate very well to the GLP-1 receptor expression pattern described by Merchenthaler *et al.* [17] in rat.

However, whilst Merchenthaler *et al.* report a moderate level of GLP-1 receptors within the caudal hippocampus of rat, no innervation of this area is seen in mouse [22^•^]. Given that effects of exogenous GLP-1 injection into the hippocampus have been observed [27,28] this raises the question of whether these receptors are activated under physiological conditions, and if so where would the GLP-1 originate from? One possible origin could be microglia involved in the response to inflammation of the brain. It has been reported that activated microglia express GLP-1, at least in culture [29]. The physiological relevance of this GLP-1 expression, though, remains to be established.

In conclusion, these anatomical findings suggest that projections from PPG neurons are appropriately placed to elicit effects on the vast majority of GLP-1 receptors expressed in the CNS.

### Central application of GLP-1 receptor antagonists to explore the role of endogenous GLP-1 in brain

Most studies to date addressing the physiological role of central GLP-1 receptors have employed intracerebroventricular (i.c.v.) injections of high concentrations of GLP-1 or a GLP-1 receptor agonist. These studies have revealed a plethora of responses, such as suppression of food intake, improved blood glucose levels, nausea, increased taste aversion, alterations in blood pressure and heart rate, hypothermia, neuroprotection and effects on learning and memory [15,28,30–35].

Whilst these studies demarcate the potential scope for effects of endogenous GLP-1 released from PPG neurons, the question remains, whether some of the effects observed are due to supraphysiological concentrations of GLP-1 producing a pattern of GLP-1 receptor activation that would not occur under physiological conditions. This question is best addressed by the injection of a GLP-1 receptor antagonist into brain in the absence of any exogenous GLP-1 or GLP-1 analogue.

In their landmark paper, more than 15 years ago, Turton and colleagues [36^••^] demonstrated that not only does i.c.v. injection of GLP-1 produce a reduction in food intake in rat but also that i.c.v. injection of the truncated exendin fragment (9–39; Ex9), a GLP-1 receptor antagonist, strongly increased food intake and body weight in satiated animals whilst having no effect on starved rats [36^••^,37] (see also [38]). This demonstrated not only a physiological role for endogenous GLP-1, but also showed that endogenous release varies with the animal's feeding state.

Several GLP-1 receptor antagonists [39] have been used to explore the role of endogenous GLP-1 in brain. Most of these studies have focused on food intake, showing that central administration of GLP-1 receptor antagonists can cause increased feeding in stressed animals, attenuate c-fos expression in the brainstem and decrease LiCl-induced anorexia and stress hormone levels [40–43]. Others have shown reduced glucose tolerance [44^•^] or impaired the insulin-dependent suppression of hepatic glucose production [45] when central GLP-1 receptors were blocked. However, whilst these studies indicate a physiological role for endogenous GLP-1 in brain, i.c.v. injection of these antagonists has precluded dissection of the specific neuronal populations involved. To overcome this limitation, several recent papers used site-specific injections into the brain parenchyma to allow the examination of specific brain nuclei [19^••^,20^••^,38,46,47^••^].

### Delineating ascending pathways: injection of antagonists into forebrain sites

**Figure 1:**
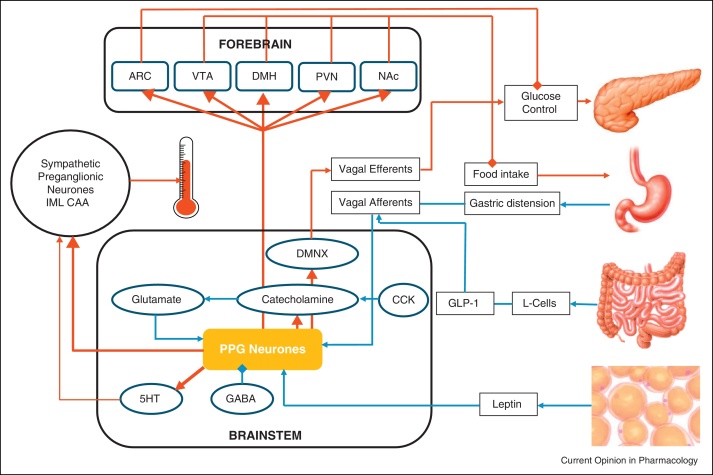
Physiologically relevant inputs to and projections from brainstem PPG neurons. Brainstem PPG neurons receive inputs (blue) related to short-term and long-term energy status. Inputs include electrical satiety signals via the vagal nerve from the stomach and gut, hormonal signals like CCK from the gut, or leptin from adipose tissue. Outputs (red) from these neurons are directed towards various forebrain sites with emphasis on food intake and glucose control. Local and descending outputs from these neurons travel to dorsal vagal efferent neurons, serotonergic (5-HT) neurons and catecholaminergic neurons in the NTS and ventrolateral medulla. These outputs might be involved in the regulation of blood glucose in the case of vagal neurons and thermoregulation for 5-HT neurons. Additionally, there are strong direct projections from PPG neurons to sympathetic preganglionic neurons in the central autonomic area (CAA) and the intermediolateral cell column (IML) in the spinal cord. ARC, arcuate nucleus; VTA, ventral tegmental area; DMH, dorsomedial hypothalamus; PVN, paraventricular nucleus; NAc, nucleus accumbens; DMNX, dorsal motor nucleus of the vagus; CCK, cholecystokinin.

Most of these studies have focused on the dissection of GLP-1 effects on food intake. Schick *et al.* demonstrated that lateral hypothalamic microinjections of Ex9 significantly augmented food intake in satiated rats only [38]. More recent studies were designed to dissect suppression of metabolically driven food intake from conditioned taste aversion and from reward system driven appetite. These papers explored the influence of the nucleus accumbens (NAc) and the ventral tegmental area (VTA) known to be involved with reward and motivation [19^••^,20^••^,46,48] (Figure 1). Specific targeting of the NAc core rather than the shell region with Ex9 was shown to increase food intake up to 2 hours after animals entered the dark phase of the circadian cycle [19^••^]. Similarly, a unilateral injection of Ex9 into the VTA increased high fat diet intake in rats at 3 and 6 hours post-injection [20^••^]. Further analysis of the effects of GLP-1R blockade in the NAc core on the intake of palatable food suggested that GLP-1 receptor activation in the NAc affects meal size rather than meal frequency [46] whereas the opposite was observed for GLP-1 receptor activation in the hindbrain [47^••^].

### Descending pathways: injection of antagonists into brainstem

Early evidence that hindbrain GLP-1 receptors are involved in different aspects of food intake than those in the hypothalamus was provided by Grill *et al.* [49] who showed that lipopolysaccharide (LPS) anorexia is alleviated by blocking GLP-1 receptors accessible from the 4th, but not the 3rd, ventricle. Subsequently, it was shown that both 4th ventricular and local caudal NTS delivery of Ex9 increased food intake in satiated rats, indicating a role for hindbrain GLP-1 receptors in ‘metabolic’ food intake [47^••^]. The same study also demonstrated that the reduction in food intake caused by gastric distension is reversed by 4th ventricular Ex9, but not when caused by duodenal nutrient infusion [47^••^]. These findings suggest that it is unlikely to be duodenally released GLP-1 entering the brainstem that activates the GLP-1 receptors, but rather electrical signals (presumably vagal) from the stomach that activate PPG neurons in the NTS. These in turn release GLP-1 locally, leading to the observed effects (Figure 1).

At present, little is known about which cell types in the lower brainstem express GLP-1 receptors, and thus what downstream pathways are likely to be involved. A recent study has identified some of the cell types receiving close appositions from PPG axons within the brainstem [21^•^]. These include about 30% of cholinergic dorsal vagal neurons, a similar proportion of catecholaminergic A1/C1 and A2/C2 neurons, and the majority of serotonergic neurons in the raphe pallidus and the parapyramidal tract. These cell populations would provide both descending and ascending projections that could potentially account for effects on food intake, thermoregulation, blood pressure, heart rate, insulin release, among others.

### What inputs do GLP-1 neurons receive?

Until the development of the PPG-YFP mouse by Reimann and colleagues [23^•^], PPG neurons could only be identified post hoc by immunocytochemistry. This limited functional analysis of this cell population to the use of immunoreactivity to c-fos or equivalent markers of neuronal activation [15,50,51]. Such studies demonstrated that PPG neurons were activated by gastric distension [51], leptin [52], LiCl and oxytocin [53], placing the PPG neurons at the core of central GLP-1 effects observed in relation to these stimuli.

The PPG-YFP mouse allowed identification of PPG neurons in living tissue and in the first study that directly recorded the electrical activity of GLP-1-expressing cells, Hisadome and colleagues discovered that leptin directly depolarises these neurones in the nucleus of the solitary tract (NTS) and that, whilst these neurons do not express GLP-1 receptors, at least a proportion of PPG neurons receive monosynaptic input from the solitary tract [54^••^] (i.e. vagal afferent fibres). These results further supported the notion that gut-derived GLP-1 would act in the periphery, rather than directly on PPG neurons in order to elicit central GLP-1 release (Figure 1). A recent study on human subjects that had undergone truncal vagotomy further supports this hypothesis [55]. Subsequently, Hisadome *et al.* reported that CCK and noradrenaline increased the activity of GLP-1-expressing neurons by enhancing glutamatergic drive [56] (Figure 1). These results demonstrated that GLP-1 neuronal activity is modulated by both long-term and short-term satiety signals. It now remains to be established whether there is a separation of these neurons into discrete subpopulations that responds to either short-term or long-term signals, and similarly whether the projection targets for the individual PPG neurons correlate with the specific inputs they receive.

### Can we interfere with the function of GLP-1 neurons *in vivo*, and what are the consequences?

Finally, in order to unequivocally determine the importance of the central GLP-1 system, it needs to be completely separated from the peripheral system. There are two key questions to be answered. Firstly, are central GLP-1 receptors only accessible for CNS derived GLP-1, and secondly, do the hindbrain GLP-1 neurons fulfil this role? The pharmacological studies employing the central injection of GLP-1 receptor antagonists address these questions only partially, because whilst they demonstrate the action of endogenous GLP-1, they cannot rule out the possibility that gut-derived GLP-1 is responsible for the observed effects on central GLP-1 receptors. Similarly, the global knockout of either the glucagon gene, or the GLP-1 receptor gene, affects both central and peripheral systems. Additionally, such a genetic approach is prone to developmental compensation. To circumvent such problems, Barrera *et al.* [57^••^] employed RNA interference, delivered by stereotaxic injection of lentivirus into the NTS, to knock down GLP-1 expression. With this approach they achieved a reduction of preproglucagon mRNA levels by 50% in NTS and by 30% in the PPG cell terminals in PVN. They observed hyperphagia and weight gain compared to control animals that received injections of scrambled shRNA. However, these controls only regained preoperative weight 28 days after surgery. Nevertheless, these data indicate that endogenous GLP-1 derived from PPG cells has a physiological role in the regulation of energy balance and it is experiments like these that will hopefully give us a more complete and detailed picture of the physiological importance of the PPG neurons over the coming years.

## Conclusions

We suggest that the effects of *neuropeptide* GLP-1 (released by PPG neurons) are distinct from the effects of *incretin* GLP-1 (released by enteroendocrine cells) and that the PPG neurons constitute a central signalling network that integrates peripheral and central signals for both long and short term nutritional and digestional status. GLP-1 neurons might produce an output signal to feeding and autonomic circuits which optimises digestion and assimilation of nutrients and regulates calorific intake.

## References and recommended reading

Papers of particular interest, published within the period of review, have been highlighted as:

• of special interest
•• of outstanding interest
